# Cost-effectiveness of severe acute malnutrition treatment delivered by community health workers in the district of Mayahi, Niger

**DOI:** 10.1186/s12960-024-00904-1

**Published:** 2024-03-29

**Authors:** Elisa M. Molanes-López, José M. Ferrer, Abdias Ogobara Dougnon, Abdoul Aziz Gado, Atté Sanoussi, Nassirou Ousmane, Ramatoulaye Hamidou Lazoumar, Pilar Charle-Cuéllar

**Affiliations:** 1 Department of Statistics and Operational Research, Faculty of Medicine, Universidad Complutense de Madrid (UCM), 28040 Madrid, Spain; 2 Department of Statistics and Operational Research, Faculty of Medicine, Interdisciplinary Mathematics Institute, Universidad Complutense de Madrid (UCM), HUMLOG Research Group, 28040 Madrid, Spain; 3 Action Against Hunger. West and Central Africa Regional, 29621 Dakar, Senegal; 4 Action Against Hunger, BP 11491, Niamey, Niger; 5 Nutrition Direction, Ministry of Health, 623 Niamey, Niger; 6 Centre de Recherche Médicale et Sanitaire (CERMES), 10887 Niamey, Niger; 7 Action Against Hunger, C/Duque de Sevilla No. 3., 28002 Madrid, Spain

**Keywords:** Severe acute malnutrition (SAM), Community health workers (CHWs), Community-based Management of Acute Malnutrition (CMAM), Cost-effectiveness, Niger, Decision analysis network (DAN), OpenMarkov

## Abstract

**Background:**

A non-randomized controlled trial, conducted from June 2018 to March 2019 in two rural communes in the health district of Mayahi in Niger, showed that including community health workers (CHWs) in the treatment of severe acute malnutrition (SAM) resulted in a better recovery rate (77.2% vs. 72.1%) compared with the standard treatment provided solely at the health centers. The present study aims to assess the cost and cost-effectiveness of the CHWs led treatment of uncomplicated SAM in children 6–59 months compared to the standard national protocol.

**Methods:**

To account for all relevant costs, the cost analysis included activity-based costing and bottom-up approaches from a societal perspective and on a within-trial time horizon. The cost-effectiveness analysis was conducted through a decision analysis network built with OpenMarkov and evaluated under two approaches: (1) with recovery rate and cost per child admitted for treatment as measures of effectiveness and cost, respectively; and (2) assessing the total number of children recovered and the total cost incurred. In addition, a multivariate probabilistic sensitivity analysis was carried out to evaluate the effect of uncertainty around the base case input data.

**Results:**

For the base case data, the average cost per child recovered was 116.52 USD in the standard treatment and 107.22 USD in the CHWs-led treatment. Based on the first approach, the CHWs-led treatment was more cost-effective than the standard treatment with an average cost per child admitted for treatment of 82.81 USD vs. 84.01 USD. Based on the second approach, the incremental cost-effectiveness ratio of the transition from the standard to the CHWs-led treatment amounted to 98.01 USD per additional SAM case recovered.

**Conclusions:**

In the district of Mayahi in Niger, the CHWs-led SAM treatment was found to be cost-effective when compared to the standard protocol and provided additional advantages such as the reduction of costs for households.

*Trial registration*: ISRCTN with ID 31143316. https://doi.org/10.1186/ISRCTN31143316

**Supplementary Information:**

The online version contains supplementary material available at 10.1186/s12960-024-00904-1.

## Introduction

Acute malnutrition is one of the major public health issues in the Sahel region. According to the World Health Organization (WHO), 38.4 million children under 5 years of age were affected by global acute malnutrition (GAM) in 2020 and of those 8 million had severe acute malnutrition (SAM) [1]. Children affected by this condition are 11 times more likely to die compared to well-nourished children [2, 3]. The Standardized Monitoring and Assessment of Relief and Transition (SMART) survey conducted in Niger in 2022 showed a GAM prevalence of 13.6% (95% CI 11.2–16.4) in the Maradi region of which 3.9% (95% CI 2.5–6.1) SAM and 9.7% (95% CI 7.6–12.3) moderate acute malnutrition (MAM) [4]. These figures mean that 457 200 children aged 6–59 months suffered from SAM in 2021 [5].

According to the Community management of acute malnutrition (CMAM) protocol, children suffering from uncomplicated SAM are treated at health centers (HCs), where they receive outpatient treatment with ready-to-use therapeutic food (RUTF) and systemic treatment with amoxicillin (50–100 mg/kg/day twice a day for 5 days) and one single dose of 500 mg of mebendazole at the first visit for deworming. In addition, they receive RUTF every visit throughout the next 6–8 consecutive weeks of follow-up. The Simplified Lot Quality Assurance Sampling Evaluation of Access and Coverage (SLEAC) survey conducted in 2016 showed a treatment coverage of 41.5% in the Maradi region. This assessment outlined several geographical barriers, especially during the hunger gap, when families deplete their food reserves and new crops have not yet been harvested. The challenges include the significant time caregivers spend traveling to or waiting at HCs, misunderstandings about malnutrition, and a lack of funds for transportation. These factors are identified as the primary obstacles contributing to low access to health services [6]. To address this issue, between 2018 and 2019, a research study was conducted to assess the effectiveness and treatment coverage by incorporating community health workers (CHWs) into health posts (HPs) in addition to the standard SAM treatment provided solely at HCs. The control group received outpatient treatment for uncomplicated SAM by nurses at HCs, while the intervention group received outpatient treatment for uncomplicated SAM by nurses at HCs or by CHWs at HPs. The primary treatment outcome was recovery defined as the absence of bilateral pitting edema (fluid build-up in feet, legs, hands and arms) for 14 days and weight-for-height *z*-score (WHZ) ≥ −2 and/or mid upper arm circumference (MUAC) ≥ 125 mm, during two consecutive follow-up visits. The results showed a statistically significant difference in recovery rates with 77.2% children recovered in the intervention group (73.1% at HCs and 83.7% at HPs) vs. 72.1% in the control group (*p* < 0.001); and a treatment coverage of 61.2% in the intervention group compared to 43.6% in the standard treatment group [7]. The CHWs-led treatment approach, part of the simplified approaches supported by UNICEF [8], has also shown its effectiveness and positive impact on coverage in other contexts such as Mali, Mauritania and Tanzania [9–11].

To plan and implement at scale, policymakers need stronger evidence to support the promising cost-effectiveness of using CHWs in child health-related settings, such as in the case of SAM treatment [12]. Bringing healthcare delivery closer to families through CHWs directly reduces the time and cost of every medical visit for the household and it is expected that it will also cause children to begin to be treated in better conditions, increasing the probability of recovery and/or reducing the duration of treatment. A study in Mali showed a recovery rate of 94.2% in the intervention group vs. 88.2% in the standard protocol highlighting that the cost per child recovered from SAM with the CHWs-led approach was 259 USD vs. 501 USD of the standard HCs-based treatment protocol (2016 USD). Each week of treatment, households under the CHWs-led approach spent half of the time receiving treatment and three times less money compared to those receiving treatment solely at the health center [13]. In Pakistan, the centralization of acute malnutrition treatment with lady health workers (CHWs in the country) did not show evidence of being a cost-effective intervention. The recovery rate was 76.0% and 83.0% in the intervention and control group, respectively and the cost per child recovered by implementing lady health workers was similar to cost at HCs (382 vs. 363, in 2016 USD). However, the cost for households receiving SAM treatment at HCs was double than the cost of care provided by lady health workers [14]. This wide variation in results suggests that cost-effectiveness may be influenced not only by the service delivery model of treating acute malnutrition in the community, but also by other factors such as the burden of acute malnutrition and the expected number of children suffering from the disease; and the quality of care and number of children recovered due to treatment delivered by these CHWs [15]. According to the Global Action Plan against Child Wasting, it is crucial to further analyze the cost-effectiveness of interventions to increase treatment coverage and achieve a reduction of the prevalence of wasting to less than 5% by 2025 [16].

The present study aims to analyze the costs and cost-effectiveness of SAM treatment delivered by CHWs compared to the standard protocol from a societal perspective in the Mayahi district of the Maradi region in Niger. This economic evaluation will be conducted on a within-trial time horizon for both cost and effectiveness results, and following the Consolidated Health Economic Evaluation Reporting Standards (CHEERS) guidelines [17] (see Additional file 1).

## Methodology

### Description of the context and intervention

**Fig. 1:**
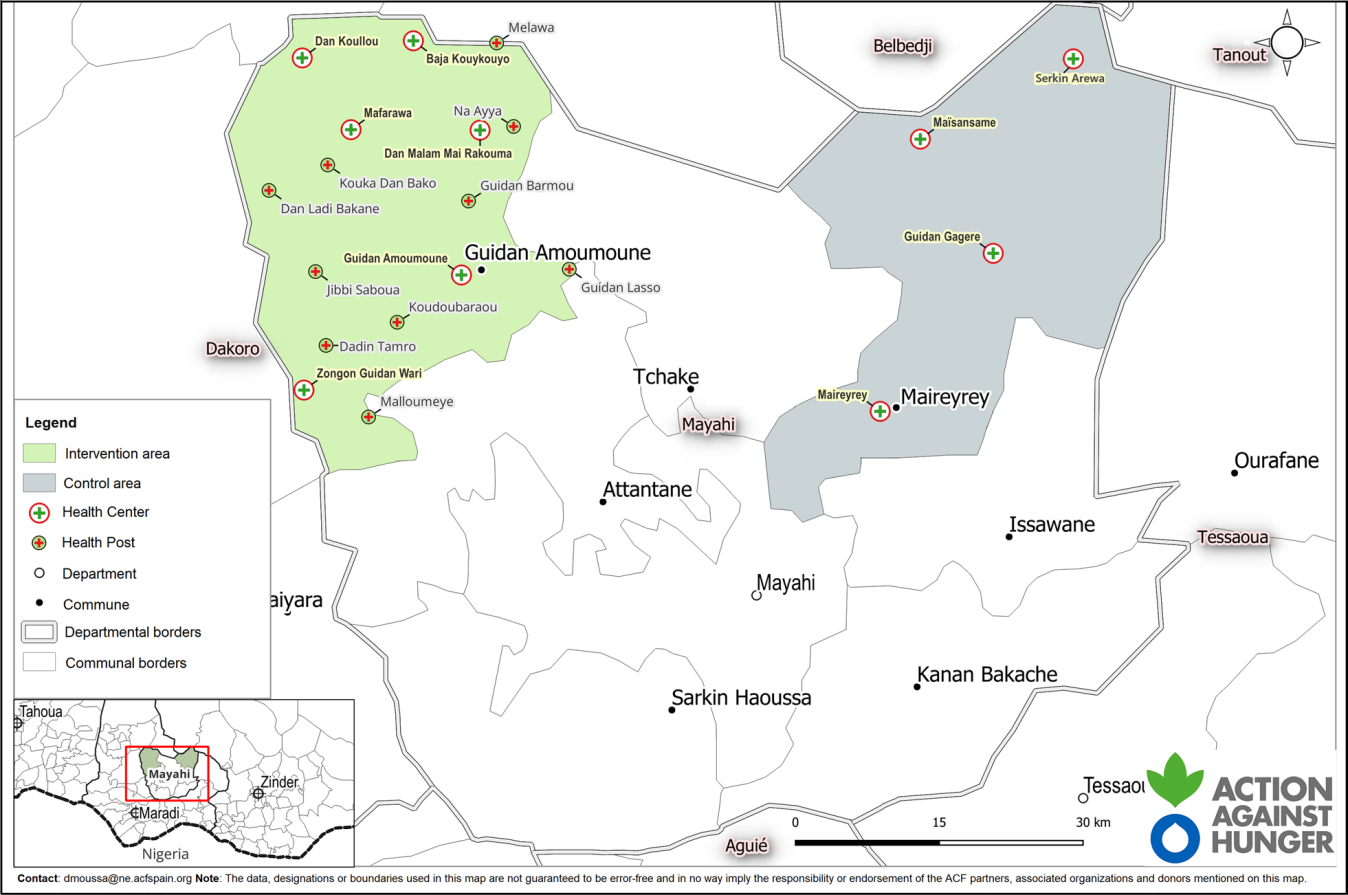
Sanitary map of the two study areas

A non-randomized controlled trial was conducted from June 2018 to March 2019 in the health district of Mayahi, of the region of Maradi in Niger. It included two rural communes, Maireyrey (the control area) and Guidan Amoumoune (the intervention area). According to the 2012 population census [18], Maireyrey and Guidan Amoumoune had, respectively, 64 183 and 88 199 inhabitants. Figure 1 shows a map of the two study areas with the location of the HCs and HPs. Socio-demographic characteristics in both areas were similar, for example family sample size, proportion of women, source of water in the household, HCs as the first option for caring children, among others. The intervention group appeared to have houses with better roofing and reported distance to HCs as the main barrier to health access [7].

Prior to the start of the study, treatment of acute malnutrition was carried out by nurses at HCs, decentralized treatment at HPs with CHWs not being allowed by the administrators of the country’s SAM policy. All children 6–59 months who attended HCs or HPs and met the inclusion criteria were recruited in the study. The inclusion criteria were the presence of mild (+) or moderate (++) edema and/or a WHZ less than − 3 and/or a MUAC less than 115 mm [19]. Cases with severe edema, medical complications, or failed appetite tests were excluded from the study and referred for inpatient treatment. Outpatient treatment for uncomplicated SAM was provided for a maximum of 8 weeks (initial visit plus seven follow-up weekly visits) by nurses at the 4 existing HCs in the control group (standard treatment), and by nurses at the 6 existing HCs and 10 additional CHWs located at HPs in the intervention group (CHWs-led treatment). At the end of the treatment, the final nutritional recovery status of the children is assessed. The Ministry of Health provided treatment, UNICEF supplied RUTF while Action against Hunger (AAH) supervised activities.

### Data collection

Treatment outcome data were obtained from the primary study [7]. Field data collection for the economic component was conducted between June 2019 in Niamey and August 2019 in Mayahi. Data on cost and resource usage were collected from (1) nurses providing SAM treatment at HCs; (2) CHWs at HPs; (3) AAH staff and partners involved in support, supervision, management and logistics; (4) caregivers of SAM children.

Financial and accounting costs of AAH Niger and study financial records were used as primary data sources. HCs staff, CHWs and project key informants including AAH staff and relevant partners were selected through deliberate sampling and interviewed using semi-structured interviews to map activities and to allocate the time for their implementation.

A total of 18 semi-structured interviews were carried out which included five CHWs, five nurses responsible for the HCs, one regional nutrition focal point, one Chief District Medical Officer, one financial District Officer, one doctor from the Ministry of Health responsible for inpatient treatment of SAM, and four AAH staff comprising a supervisor and the heads of the finance, logistics and human resources (HR) departments in Niamey and Maradi. These interviews allowed us to identify the resources used for the treatment of SAM at the HCs and HPs including but not limited to RUTF, drugs, medical equipment and consumables. Moreover, 16 focus group discussions were conducted: seven with community volunteers and nine involving children’s caregivers, who were enabled to gather data on the costs incurred by households for seeking care, and on the associated opportunity costs resulting from loss of income.

The tasks and activities linked to the provision of treatment such as consultations, training, and supervision of CHWs were mapped out and the corresponding time allocation for each activity was collected. Costs were calculated triangulating both accounting records and information obtained through key informant interviews.

### Data analysis

#### Costs

The cost analysis is conducted using a combination of activity-based costing and a bottom-up approach, in line with the classification proposed by Njuguna et al. [20] on a within-trial time horizon. Besides, a societal perspective is employed to assess the impact of incorporating CHWs on household costs. Consequently, since opportunity costs associated with family income losses are included, our analysis considers economic and not just financial costs [21].

The allocation of fixed costs to activities is determined using activity-based costing, and the specific details are provided in Table 1. Fixed costs include the costs independent of the number of children admitted for treatment and comprise activities grouped in the following categories: Supervision, Staff support and HPs implementation. In addition, HPs implementation category includes the following subcategories: Management and coordination, Training, HPs procurement and RUTF logistics. On the one hand, Supervision and Staff support categories’ costs are common to the entire program, and they must be distributed between control and intervention groups according to the population size of the areas. An exception was considered in the Monthly monitoring activity of the Supervision category as the number of supervisors involved differed. In specific, the control group had one supervisor while the intervention group had two, resulting in a double cost for the intervention group in this activity. On the other hand, all costs in the HPs implementation category are allocated entirely to the intervention group.

**Table 1:** Input costs per activity and comparison by study group

Cost input	Payer	Control	%	Intervention		%
Total costs		64,433.05	100	163,721.71		100
Total fixed costs		14,994.07	23.3	77,897.12		47.6
Supervision		7,119.41	11.0	12.981,97		7.9
Monthly monitoring	AAH	5,111.10		10,222.19		
District nutrition supervision	AAH	439.51		603.97		
Regional nutrition supervision	AAH	264.92		364.04		
National nutrition supervision	AAH	1,303.88		1,791.77		
Staff support		7,874.67	12.2	10,821.21		6.6
Support Mayahi	AAH	86.75		119.21		
Support Niamey	AAH	3,982.65		5,472.88		
Logistic Mayahi	AAH	99.14		136.24		
Logistic Niamey	AAH	224.02		307.85		
Finances Mayahi	AAH	27.88		38.32		
Finances Niamey	AAH	597.40		820.93		
Project manager	AAH	146.50		201.32		
National nutrition direction	MoH	439.51		603.97		
Regional nutrition direction	MoH	586.01		805.29		
District nutrition direction	MoH	1,684.79		2,315.21		

				HCs	HPs	
HPs implementation		–		0.00	54,093.93	33.1
Management and coordination		–		–	15,130.43	9.2
Meeting support	AAH	–		–	9,565.22	
Community volunteers’ motivation	AAH	–		–	5,565.22	
Training		–		–	4,900.35	3.0
CHWs training	AAH	–		–	2,869.57	
CHWs training supervision	AAH	–		–	1,100.35	
Car rental	AAH	–		–	930.43	
HPs procurement		–		–	30,287.06	18.5
Refurbishment	AAH	–		–	11,100.00	
CHWs equipment	AAH	–		–	3,860.87	
Materials and consultation records	AAH	–		–	14,395.76	
Equipment and materials transport	AAH	–		–	930.43	
RUTF logistics		–		–	3.776.09	2.3
Transport	AAH	–		–	2,328.26	
Stock follow-up tools	AAH	–		–	1,447.83	
Total variable costs		49,438.98	76.7	57,364.65	28,459.94	52.4
Transport	HH	18.354,78	28.5	13.179,13	6,365.22	11.9
Opportunity costs	HH	3,441.52	5.3	4,942.17	–	3.0
RUTF procurement	UNICEF	22,353.91	34.7	31,662.26	20,287.04	31.7
Healthcare delivery HR	MoH	3,441.52	5.3	4,942.17	884.06	3.6
Hospital referral	MoH /HH	1,847.24	2.9	2,638.91	923.62	2.2

Costs in January 2019 US dollars

*HC* Health center, *HP* health post, *CHW* community health worker, *RUTF* ready-to-use therapeutic food, *AAH* Action Against Hunger, *MoH* Ministry of Health, *HH* households, *HR* human resources

The bottom-up approach is used to compute variable costs, which are those dependent on the number of children admitted for treatment and/or the number of medical visits attended. Variable costs include the following categories: Transport, Opportunity costs, RUTF procurement, Healthcare delivery HR and Hospital referral. In each of these categories, the unit cost is multiplied by the number of medical visits attended, except for the Hospital referral category. In this case, the unit cost (which includes transport, care during inpatient treatment and opportunity cost for families) is multiplied by the number of children admitted for treatment but later transferred to the hospital due to medical complications developed during the follow-up. To determine the total cost of transport, opportunity and healthcare delivery HR for each group, the initial and final visits are added to the follow-up visits per each child. RUTF procurement total costs were calculated considering the initial and follow-up visits but excluding the final visit. Table 1 also outlines which parties are responsible for the costs associated with each activity and/or category.

Research costs related to investigator salaries and study registration are not included. All costs are reported in CFA Francs and converted to US Dollars using the January 2019 exchange rate (1 US Dollar = 575 CFA Francs). Since all costs were measured within a 1-year period, no discounting or inflation adjustments are applied, and it is assumed that no capitalization has occurred.

#### Cost-effectiveness

**Fig. 2:**
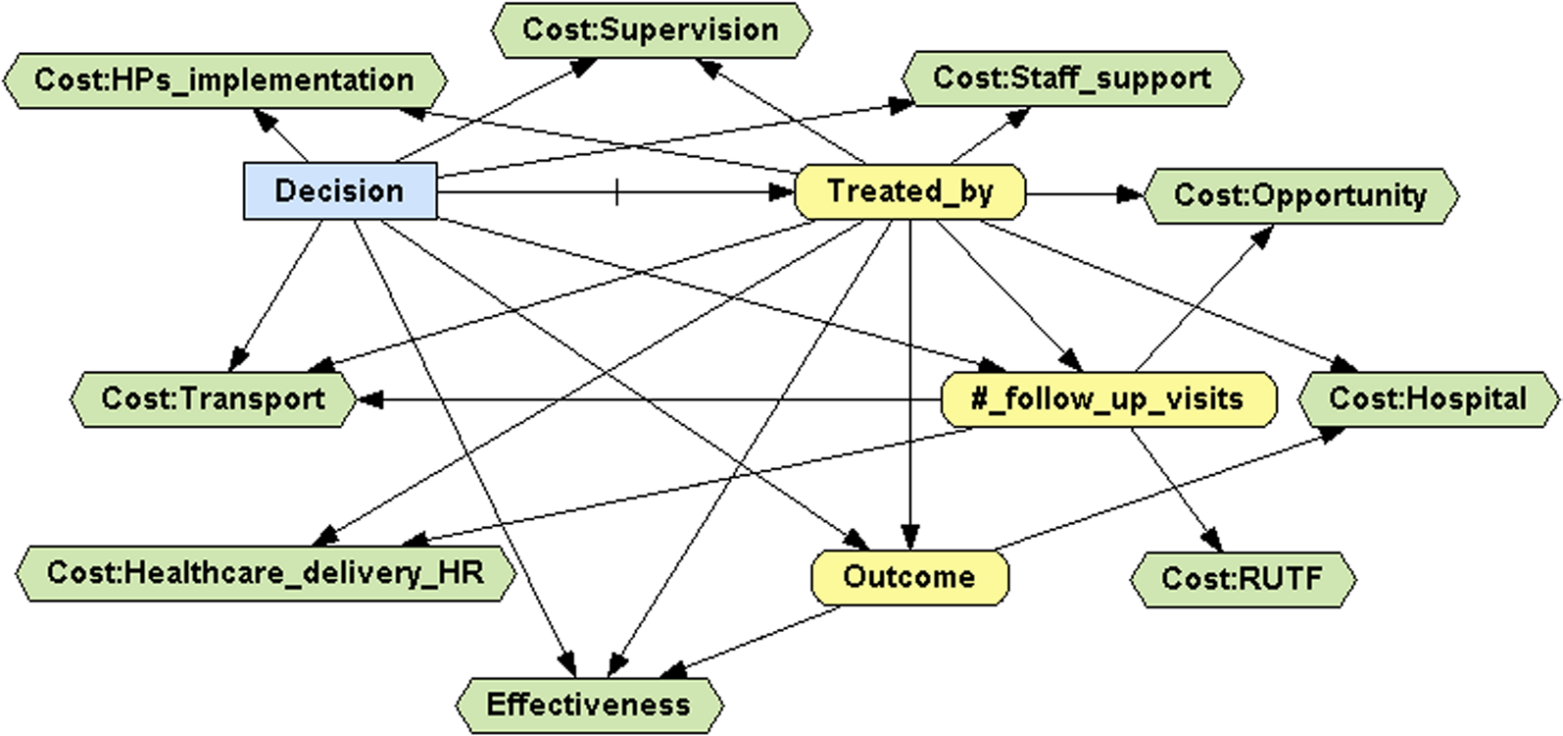
Decision analysis network

To carry out the cost-effectiveness analysis of our data, the decision analysis network (DAN) presented in Figure 2 was developed in OpenMarkov (version 0.4.0), an open-source software package for probabilistic graphical models (PGMs), developed by the Research Centre for Intelligent Decision-Support Systems (CISIAD) at the Universidad Nacional de Educación a Distancia (UNED) in Madrid, Spain [22–24]. OpenMarkov has recently been applied in several medical cost-effectiveness analyses [25–27]. More specific details are given in Additional file 2.

Based on this DAN, two approaches were considered. In the first approach, the measure of effectiveness was the recovery rate and the cost was measured per child admitted for treatment, as in the works of Rogers et al. [13, 14]. Note that both cost and effectiveness measures are normalized to a per-patient basis. In the second approach, the measures of effectiveness and cost were, respectively, the number of children recovered and the total cost, as in the works of Johns et al. [28] and Wilunda et al. [11]. An advantage of this approach is that it considers the increased coverage attained when the treatment is delivered by CHWs at HPs, thus effectively addressing barriers to accessing health care. To ensure the fairness of the comparison, the total cost and the number of children recovered from the intervention area were rescaled to the population size of the control area, as was done previously by Zeng et al. [29, 30]. To compare the control and intervention treatments in terms of cost-effectiveness, the average cost-effectiveness ratio (ACER) of each treatment and the incremental cost-effectiveness ratio (ICER) were calculated [31].

#### Sensitivity analysis

A multivariate probabilistic sensitivity analysis based on 1000 Monte Carlo simulations was performed under each approach to assess how changes in the input data affected the base case results. Dirichlet distributions were used for probabilities (three yellow nodes in Fig. 2), assuming standard deviations less than 0.1. Triangular symmetric distributions were used for those costs that were independent of the number of follow-up visits (Cost:Supervision, Cost:Staff_support, Cost:HPs_implementation and Cost:Hospital nodes), with the interval endpoints set at 10% from the mode. In contrast, normal distributions were used for those variable costs that were dependent on the number of follow-up visits (Cost:Transport, Cost:Opportunity, Cost:RUTF and Cost:Health_delivery_HR nodes), with standard deviations equal to 10% of the corresponding means. Illustrative examples of how uncertainty was introduced into the DAN presented in Fig. 2, depending on the nature of each node, are given in Additional file 3.

## Results

### Costs

Table 1 presents the input costs for the base case, along with the relative percentage of each cost category and subcategory by group.

The transport cost per visit was 3.48 USD (2 h round trip on average) in the control group while in the intervention group it varied from 1.74 USD (1 hour round trip on average) for children treated at HCs to 1.30 USD (45-min round trip on average) for children treated at HPs. The opportunity cost per visit for children treated at HCs was 0.65 USD in both the control group and the intervention group, while for children treated at HPs this opportunity cost was reported by focus group participants to be null. The cost of the healthcare delivery HR was 0.65 USD per visit for children treated by nurses at HCs and only 0.18 USD for children treated by CHWs at HPs. To obtain these values, we have taken into account that visits lasted on average 20 min and that the monthly salaries of nurses and CHWs were, respectively, 313.04 USD and 86.96 USD. The RUTF procurement cost was 4.96 USD per visit across all groups. The unit cost per child transferred to the hospital is 131.95 USD in both groups. In the control group, 14 children were transferred, while in the intervention group, 27 children were transferred, with 20 treated at HCs and 7 at HPs.

The group frequencies and percentages of the follow-up visits are presented in Table 2. Note that children with 0 follow-up visits are those who were admitted for treatment in the initial visit but did not attend any follow-up visits for some reason.

**Table 2:** Distribution of the number of follow-up visits per child

#Follow-up visits	Control		Intervention			
			HCs		HPs	
	# Children	%	# Children	%	# Children	%
0	2	0.26	32	2.69	16	2.03
1	26	3.39	37	3.11	26	3.30
2	64	8.34	116	9.75	85	10.80
3	50	6.52	168	14.12	133	16.90
4	88	11.47	222	18.66	178	22.62
5	169	22.03	214	17.98	151	19.19
6	334	43.55	340	28.57	142	18.04
7	34	4.43	61	5.13	56	7.12
**Total**	767	100	1190	100	787	100

*HC* Health center, *HP* health post

### Cost-effectiveness

Table 3 presents the base case cost-effectiveness results. According to the first approach, the recovery rate was 72.1% in the control group and 77.2% in the intervention group [7]. The average cost per child admitted for treatment was 84.01 USD in the control group and 82.81 USD in the intervention group. These results showed that the CHWs-led treatment dominates the standard treatment, since it provided better outcomes according to both indicators. However, according to the second approach, the CHWs-led treatment was not only more effective than the standard treatment but also more expensive. The ACER, calculated following either approach, was 116.52 USD per child recovered in the control group and 107.22 USD in the intervention group.

**Table 3:** Cost-effectiveness results

	Control	Intervention	Intervention rescaled
# children admitted for treatment	767	1,977	1,438.68
# children recovered	553	1,527	1,111.21
Recovery rate	72.1%	77.2%	77.2%
Total cost	64,433.05	163,721.71	119,141.38
Cost per child admitted for treatment	84.01	82.81	82.81
Cost per child recovered	116.52	107.22	107.22
ICER			98.01

Costs in January 2019 US Dollars.

*ICER* incremental cost-effectiveness ratio

As part of the second approach, the ICER was calculated at 98.01 USD, implying that having one additional child recovered in the rescaled intervention group required an additional cost of 98.01 USD compared to the control group. The rescaling factor was 0.7277 (64,183/88,199) while the ICER value of 98.01 USD was obtained from the results collected in Table 3:

$$ICER = \frac{\mathrm{119,141.38}-\mathrm{64,433.05}}{\mathrm{111,121}-553}=98.01,$$

where $\mathrm{119,141.38}=0.7277\times \mathrm{163,721.71}$ and $\mathrm{1,111.21}=0.7277\times 1527.$ This result signifies that, compared to the control group, approximately 558 more children recovered in the rescaled intervention group at an additional cost of 54,708.33 USD.

### Sensitivity analysis

**Fig. 3:**
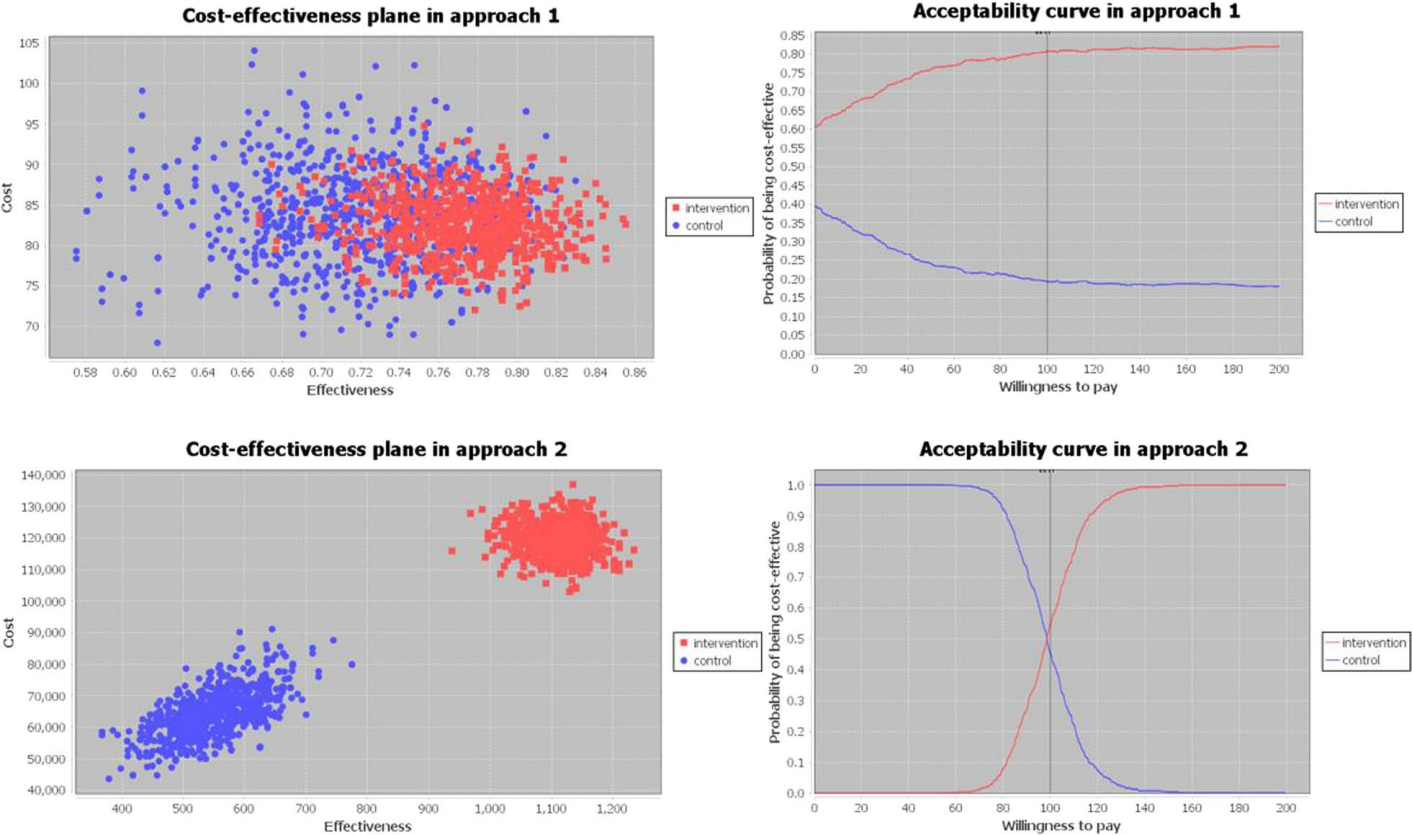
Sensitivity analysis graphs

Figure 3 shows the results of the multivariate probabilistic sensitivity analysis carried out under each approach. In the cost-effectiveness plane, each pair of blue and red points represents the cost and the effectiveness corresponding to the control group (in blue) and the intervention group (in red) of one Monte Carlo simulation. This cost-effectiveness plane provides a clear image of the uncertainty introduced in the input data. Greater concentration of points indicates reduced uncertainty, whereas increased scattering of points indicates greater uncertainty. The cost-effectiveness plane allows the calculation of the percentage of simulations where one treatment is cost-effective compared to the other based on a specific willingness to pay (WTP) value. Interestingly, under the first approach, the probability of being cost-effective is always higher for the intervention group than the control group, independently of the WTP value. However, this changes under the second approach, where (1) for a WTP value smaller than 98.01 USD, which coincides with the ICER of the base case, the standard treatment has more probability of being cost-effective than the CHWs-led treatment; (2) for values above 98.01 USD the probability increases for the CHWs-led treatment; and (3) when the WTP value was 98.01 USD, both treatments have the same probability of being cost-effective.

## Discussion

This analysis showed that CHWs-led treatment in Niger is a cost-effective intervention, compared to the standard protocol delivered solely at HCs, which is consistent with the findings from previous studies in other contexts. In terms of costs, RUTF procurement was the category with the highest cost, representing 34.7% of the total cost in the control group and 31.7% in the intervention group. This proportion was similar to the one obtained in Malawi [32], lower than in Tanzania [11], higher than Pakistan, where cost related to RUTF represents 15.2% in the control and 15.7% in the intervention group [14], and much higher than Mali, where the cost is 6.0% and 11.8% in the control and intervention group, respectively [13]. Transport was the second highest cost category for the control group reaching 28.5% but represented only 11.9% of the total cost for the intervention group. This difference can be explained by three main factors. First, the location of HPs and therefore the reduced distance to health services in the intervention area compared to the control area. Second, the fixed costs being much higher in the intervention group (47.6%) compared to the control group (23.3%) due to the implementation of the HPs, and consequently, the variable costs categories, such as Transport, having less relative relevance for the intervention group than for the control group. Third, as presented in Table 2, children in the control group required more follow-up visits to reach recovery than those in the intervention group (4.88 vs. 4.30 on average). In specific, the study found a higher number of children in the control group who needed to attend at least 6 follow-up visits to be discharged as recovered. The most plausible explanation for the difference in the number of visits among groups is that children from the control group accessed treatment later and in a worse clinical condition [7]. This finding supports the hypothesis that CHWs facilitate early identification and treatment of children leading to a shorter average length of stay and, consequently, reducing the variable costs, including the transport cost [33]. Regarding the costs of the healthcare delivery HR, they constitute less than 6% of total costs in both groups. However, these costs represent a lower percentage in the intervention group (3.6%) than in the control group (5.3%).

The costs were distributed among the different payers as follows: 11.9% by the Ministry of Health, 53.8% by NGOs and 34.3% by the households in the control group; 7.7% by the Ministry of Health, 77.0% by NGOs and 15.3% by the households in the intervention group. NGOs incurred the highest cost in both groups while communities incurred the lowest costs, which aligns with the findings of other studies conducted previously [13, 14]. The cost to implement the intervention increased the NGOs’ cost percentage in the intervention group compared to the control group.

In our study, the costs per child admitted to treatment (82.81 USD) and recovered (107.22 USD) in the intervention group are among the lowest reported in programs where CHWs support the treatment of SAM in Africa. For example, these costs were 166.31 USD and 179.40 USD in Ethiopia [34], 146.50 USD and 161.77 USD in Tanzania [11] and 259.91 USD and 275.89 USD in Mali [13]. This lower cost in our study could be influenced by the higher number of children admitted in Niger, which is 1,977, with fixed costs being shared. In the three other studies, less than 400 children under five were admitted in the intervention group. However, although we have expressed all these costs in 2019 US dollars, comparing CMAM programs can be challenging due to the differences in methodologies, timelines, ways of implementation and data collection.

In Niger, the treatment of acute malnutrition is free of charge for communities. However, during the treatment community members incur expenses linked to transport costs to reach health services and the corresponding opportunity costs associated with seeking treatment. Our study showed that a CHWs-led treatment decreased these expenses. The cost per child admitted for treatment in the control group amounted to 28.74 USD for the households, whereas in the intervention group it was 12.62 USD, less than half of the control group's cost. This difference slightly increases when comparing the cost for the households per child recovered (39.86 USD in the control group vs 16.34 USD in the intervention group). Regarding the cost per visit, in the control group the households that received treatment at HCs spent an average of 4.13 USD. In contrast, within the intervention group, households spent 2.39 USD per visit at HCs and 1.30 USD at HPs. These differences may be explained by the greater proximity to health services in the intervention area, which is one of the main arguments in favor of the CHWs-led treatment approach. Similar findings were presented in Mali [13], where households whose children received treatment from CHWs spent on average three times less money. In the case of Pakistan, the treatment with the lady health workers did not lead to cost savings for households [14]. This significant reduction in cost reported in our study could enable not only an increase in provision and access to health services, but also, from a societal perspective, cost savings that could free resources for other purposes, and time savings from reduced treatment, meaning that caregivers and patients can use the time for other activities.

Our findings indicate that SAM treatment delivered by CHWs is a cost-effective intervention compared to the standard treatment, with an additional cost of 98.01 USD per each additional child recovered. Implementing this program over a longer period of time could enhance its cost-effectiveness since some of the fixed costs would be diluted over time. For example, if the program had been continued long enough for the number of children admitted to double, and assuming that fixed costs had not increased, the projected cost per child admitted for treatment in the intervention (control) group would be 63.11 USD (74.23 USD), and the projected cost per child recovered would be 81.71 USD (102.96 USD). In addition, the projected ICER under the second approach would be 60.66 USD, which is 38% less than the ICER calculated in the base case. In the same way, if the program had been continued long enough for the number of children admitted to quintuple, the projected ICER would be 38.26 USD, 61% less than in the base case.

According to our first approach, in contrast with the findings reported by Rogers et al. in the Sindh Province of Pakistan [14], our intervention group had better outcomes in terms of both recovery rates and cost per child admitted for treatment compared to the control group, which was also reflected by the smaller ACER in the intervention group.

The second approach proved particularly relevant in scenarios where the new intervention effectively tackles barriers of access to healthcare, enabling a greater number of children to be admitted for treatment at earlier stages of severity, resulting in shorter recovery times and reduction in variable costs. According to this second approach, rescaling the measures of effectiveness and cost, namely the total number of children recovered and total cost incurred, based on the population sizes, ensured a fairer comparison and should be considered in similar cost-effectiveness studies. However, this aspect has been at times overlooked in existing literature.

The second approach also provided an additional advantage over the first approach by conveying a clear message, particularly valuable for policymakers and donors, regarding the additional cost necessary to achieve the recovery of an additional child through the CHWs-led treatment compared to the standard treatment. This approach considered the increased number of children admitted for treatment as well as the higher recovery rate in the CHWs-led treatment while the first approach only took into account the recovery rate, independently of the number of children admitted for treatment.

This research presents several strengths. Concerning costs, the most important is the use of a societal perspective, which incorporates household costs, emphasizing the cost savings for families resulting from the inclusion of CHWs. Besides, the combination of activity-based costing and a bottom-up approach has allowed us to calculate variable costs according to the number of children admitted for treatment and the number of follow-up visits made. Regarding the cost-effectiveness analysis, the use of two different methodological approaches has made it possible for our study to be comparable across a wider group of studies and to yield significant results that would remain hidden if only the first approach had been used.

The study also presents two important limitations. First, the data come from a non-randomized control trial, which does not allow us to assume comparability between the two groups. In addition, the potential impact of the difference in the population size between the groups has been minimized by rescaling the data to calculate the ICER. Second, some costs such as RUTF, transportation costs, community volunteer salaries and material for medical appointments were considered for HPs but not for HCs in both control and intervention groups. The absence of these costs, for which data were not available, may have slightly biased the results in favor of the control group.

Considering the substantial number of children affected by acute malnutrition every year, and the geographical, economic and social barriers to health service delivery, new approaches are necessary to increase treatment coverage. Given the limited availability of resources, it is crucial for the Niger Ministry of Health and international stakeholders to prioritize their interventions. The present study aligns with the available evidence regarding the effectiveness of the CHWs-led treatment as one of the proposed simplified approaches also showing its ability to reduce expenses for families. Policymakers can consider these results when making decisions about the implementation of this new approach to tackle the impact of acute malnutrition. 

### Supplementary Information

**Additional file 1. **CHEERS 2022 Checklist.

**Additional file 2.** Specific details of the DAN displayed in Figure 2.

**Additional file 3. **Illustrative examples of how uncertainty was incorporated in the DAN displayed in Figure 2. 

## Data Availability

The datasets used and/or analyzed during the current study are available from the corresponding author on reasonable request.
