# Supplementary material for: Cost-effectiveness of severe acute malnutrition treatment delivered by community health workers in the district of Mayahi, Niger
Source: Hum Resour Health. 2024 Mar 29;22:22. doi: 10.1186/s12960-024-00904-1 (PMC10979590; doi:10.1186/s12960-024-00904-1)
Supplement: Supplementary file 2 — Additional file 2. Specific details of the DAN displayed in Figure 2. [file 12960_2024_904_MOESM2_ESM.docx]

# **Specific details of the DAN displayed in Figure 2**

The graphical user interface of OpenMarkov allows you to build and evaluate different probability graphical models (PGMs) such as decision trees, Bayesian networks, influence diagrams, decision analysis networks (DANs) and Markov influence diagrams, among others. OpenMarkov has recently been used in a cost-effectiveness analysis of several medical applications, such as paediatric bilateral cochlear implantation [25], cone-beam computed tomography in the management of complex phalangeal fractures [26] and contrast-enhanced ultrasound in the management of pancreatic cystic neoplasms [27]. A DAN is a type of PGM for the analysis of decision problems with a much more compact representation than a decision tree and that, unlike influence diagrams, can represent asymmetric decision problems. The DAN presented in Figure 2 contains a single decision node, represented by a blue rectangle that implicitly includes the two available alternatives: control and intervention treatments. Then, three probability nodes are presented as yellow rounded rectangles. The node “Treated_by” indicates if the child is treated at an HC or at an HP. All children belonging to the control group are treated at HCs, whereas the children belonging to the intervention group can be treated at HCs or at HPs. The node “#_follow_up_visits” allows modelling and randomising the number of follow-up visits (between zero and seven) that each child attended. The node “Outcome” includes all possible treatment outcomes for each child. The green hexagons correspond to the value nodes, where the effectiveness or the costs are assigned in line with the collected data. Specifically, the nodes “Cost:Supervision”, “Cost:Staff_support” and “Cost:HPs_implementation” correspond to fixed costs and the nodes “Cost: Transport”, “Cost:Transport”, “Cost:Opportunity”, “Cost:RUTF”, “Cost:Healthcare_delivery_HR” and “Cost:Hospital” correspond to variable costs. All the cost nodes match the categories used in Table 1. The directed links of the DAN represent a causal influence. The short perpendicular single line crossing the arrow connecting the node “Decision” to the node “Treated_by” makes it possible to enforce the rule that all children in the control group are treated at HCs and none at HPs.
