# Supplementary material for: Cost-effectiveness of severe acute malnutrition treatment delivered by community health workers in the district of Mayahi, Niger
Source: Hum Resour Health. 2024 Mar 29;22:22. doi: 10.1186/s12960-024-00904-1 (PMC10979590; doi:10.1186/s12960-024-00904-1)
Supplement: Supplementary file 3 — Additional file 3. Illustrative examples of how uncertainty was incorporated in the DAN displayed in Figure 2. [file 12960_2024_904_MOESM3_ESM.docx]

# **Illustrative examples of how uncertainty was incorporated in the DAN displayed in Figure 2**

For the probability node “#_follow_up_visits”, the uncertainty was introduced based on Dirichlet distributions, Dir (α_0_, …, α_7_), with the following parameters: Dir (α_0_=0.26, α_1_=3.39, α_2_=8.34, α_3_=6.52, α_4_=11.47, α_5_=22.03, α_6_=43.55, α_7_ =4.43) for children in the control group, Dir (α_0_=2.69, α_1_=3.11, α_2_=9.75, α_3_=14.12, α_4_=18.66, α_5_=17.98, α_6_=28.57, α_7_ =5.13) for children treated at HCs in the intervention group, and Dir (α_0_=2.03, α_1_=3.30, α_2_=10.80, α_3_=16.90, α_4_=22.62, α_5_=19.19, α_6_=18.04, α_7_ =7.12 for children treated at HPs in the intervention group. For the base case, the probability of a child being admitted for treatment and requiring 6 follow-up visits was 43.55% in the control group, 28.57% at HCs in the intervention group, and 18.04% at HPs in the intervention group (see Table 2). The value of the α_6_ parameter was therefore set to 43.55, 28.57, and 18.04, respectively.

For the fixed cost node “Cost:Supervision”, the following Triangular distributions T(min, max, mode) were considered: T(min=8.35, max=10.21, mode=9.28) for children in the control group, and T(min=5.91, max=7.22, mode=6.57) for children in the intervention group. For the base case, the values set for the mode of the Triangular distributions (9.28 USD and 6.57 USD) are the supervision costs per child in the control group (7,119.41/767=9.28) and in the intervention group (12.981,97/1977=6.57).

For the variable cost node “Cost:RUTF”, defined in terms of the initial visit and the number of follow-up visits, β× (1 + “#_follow_up_visits”), a Normal distribution with parameters of 4.95652 and 0.49652 was considered for modelling the coefficient. For the base case μ=4.95652 and, consequently, the mean of 4.95652× (1 + “#_follow_up_visits”) yielded a total variable cost of 22,353.91 USD linked to RUFT procurement in the control group (see Table 1).
